# Establishment and identification of organoids from human circulating colorectal cancer cells

**DOI:** 10.1002/ctm2.247

**Published:** 2020-12-12

**Authors:** Long Zhang, Shaobo Mo, Xiang Hu, Xiaoji Ma, Weixiang Jin, David Haixiang Peng, Yu Zhou, Xiaofei Liang, Xinxin Rao, Xiaoya Xu, Zhen Zhang, Guoqiang Hua, Sanjun Cai, Yaqi Li, Junjie Peng

**Affiliations:** ^1^ Department of Colorectal Surgery Fudan University Shanghai Cancer Center Fudan University Shanghai China; ^2^ Department of Oncology Shanghai Medical College Fudan University Shanghai China; ^3^ Cancer Institute Fudan University Shanghai Cancer Center Fudan University Shanghai China; ^4^ Shanghai Epione Medlab Shanghai China; ^5^ Ju Kang (Shanghai) Biotechnology Co., Ltd. Shanghai China; ^6^ Shanghai Jujian New Cancer Technology Center Shanghai China; ^7^ Institute of Radiation Medicine, and Cancer Institute Fudan University Shanghai Cancer Center Fudan University Shanghai China; ^8^ Department of Radiation Oncology Fudan University Shanghai Cancer Center Fudan University Shanghai China

Dear Editor,

Patients diagnosed with metastatic colorectal cancer (mCRC) often suffer from unresectable metastases, which lead to a rather poor prognosis.^1^ Although efforts have been made, the need for personalized therapy for mCRC patients is still urgent. Human CRC organoids derived from CRC patients’ primary tumor samples closely recapitulate several properties of the original tumor and provide a platform for personalized therapy design.^2^, ^3^ However, the dependence of resected primary samples of PDO model limited its application in mCRC patients. Circulating tumor cells (CTCs) are tumor cells that have detached from the primary tumor and circulate in the peripheral blood of patients, which are thought to be critical for cancer metastasis.^4^ Thus, CTCs have been approved and are used in clinical trials as an important component of liquid biopsies for metastatic breast, prostate, and colorectal cancers.^5^ It has been reported that CTC organoids were established from patients with advanced prostate cancer,^6^ providing evidence for the feasibility of culturing organoids from CRC CTCs, and this inspired us to establish a CTC organoid model for future personalized therapy exploration in mCRC patients suffering from unresectable metastases.

We enrolled 26 patients diagnosed with mCRC and treated in Fudan University Shanghai Cancer Center (FUSCC) for CRC CTC organoid culture, and the baseline clinicopathological characteristics of the patients are shown in Table S1. To isolate CTCs, we used both filtration method and CD45 depletion method.^6^ For filtration method, we used a filtration set obtained from Ju Kang (Shanghai) Biotechnology Co., Ltd. The filtration set is composed of a filter and a round‐bottomed collection tube (Figure 1A). The filter is sealed at bottom with a porous filtration membrane. The diameter of each pore on the membrane is 5.5 μm (Figure 1A). About 8∼10 mL peripheral blood was acquired from each mCRC patient. The blood was added to the filter, followed by centrifugation (Figure 1B). PBS was added to the filter to flush and wash the membrane of the filter for three times. Then the filter was put on a 50‐mL centrifuge tube up‐side‐down and cold PBS was added to the lower side of the membrane to flush the remaining cells on the membrane into the centrifuge tube. After centrifugation, cells were resuspended and transferred to a 15‐mL centrifuge tube precoated with 1% BSA/PBS. After centrifugation and discarding supernatant, the tube was put in ice to cool. For CD45 depletion method, we followed the method provided by Dong Gao et al.^6^ Cells were also collected in a precoated 15‐mL centrifuge tube and cooled. To culture CTC organoids, 200 μL Matrigel was added to resuspend the precipitated cells, and 50 μL droplet was added to a well of a 24‐well plate. After Matrigel polymerization, 800 μL intestinal organoid culture medium^7^ supplemented with Y27632 (10 μM; Selleck) was added and the plate was put in a cell incubator (Figure 1B). We observed that the cells harvested using the filtration method showed similar diameters, and the background of the seeded Matrigel droplet was clean (Figure 1C). Using the CD45 depletion method, we harvested many more cells with different diameters, resulting in a relatively complex background (Figure 1D).

**FIGURE 1:**
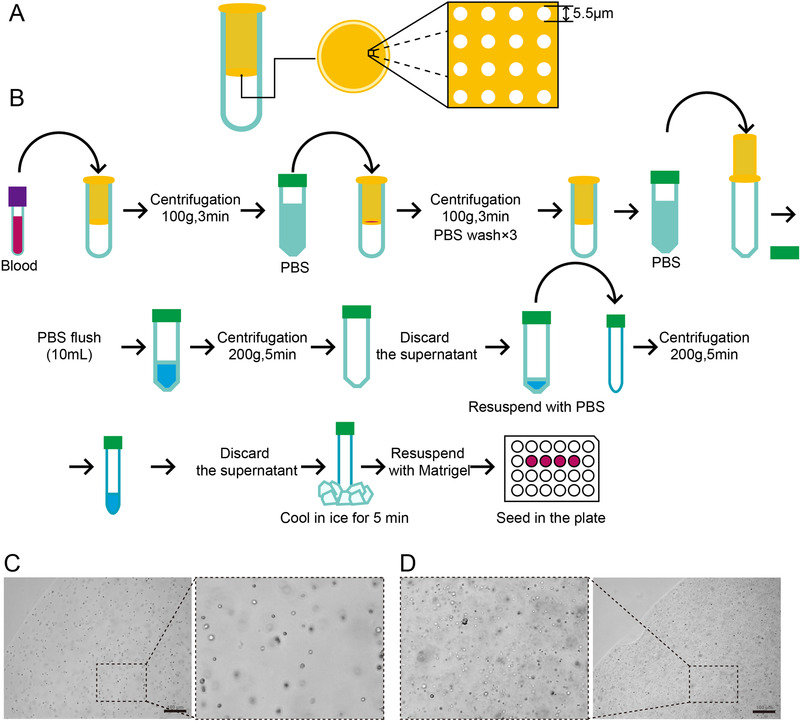
The isolation method and the seeding for circulating tumor cells. (**A**) The filtration set used in the study. The filtration set is composed of the inner filter and the outer round bottomed collection tube. The inner filter is a tube that is sealed with a porous filtration membrane at its bottom. The diameter of the pores is 5.5 μm. (**B**) The workflow of the isolation of CTCs for the cultivation of CTC organoids. **(C**) The seeded cells obtained using filtration method showed similar sizes and a clean background. (**D**) The seeded cells obtained using CD45 depletion method showed different sizes and a complex background. Scale bars = 100 μm in (**C)** and (**D)**

After approximately 14 days, the first passage of CTC organoid was observed, which showed a solid subsphaeroidal shape (Figure 2A). After passaging and culturing for approximately 14 days, there were multiple solid round CTC organoids. However, the second passage of CTC organoids were smaller than the first one, although the cultivation time was the same (Figure 2B). After the CTC organoids were passaged for the second time, it took the organoids approximately 1 month to grow into a shape resembling a mature dandelion (Figure 2C). We succeeded in cultivating 14 organoid lines out of 26 blood samples derived from mCRC patients, making the success rate 53.8% (Figure 2D).

**FIGURE 2:**
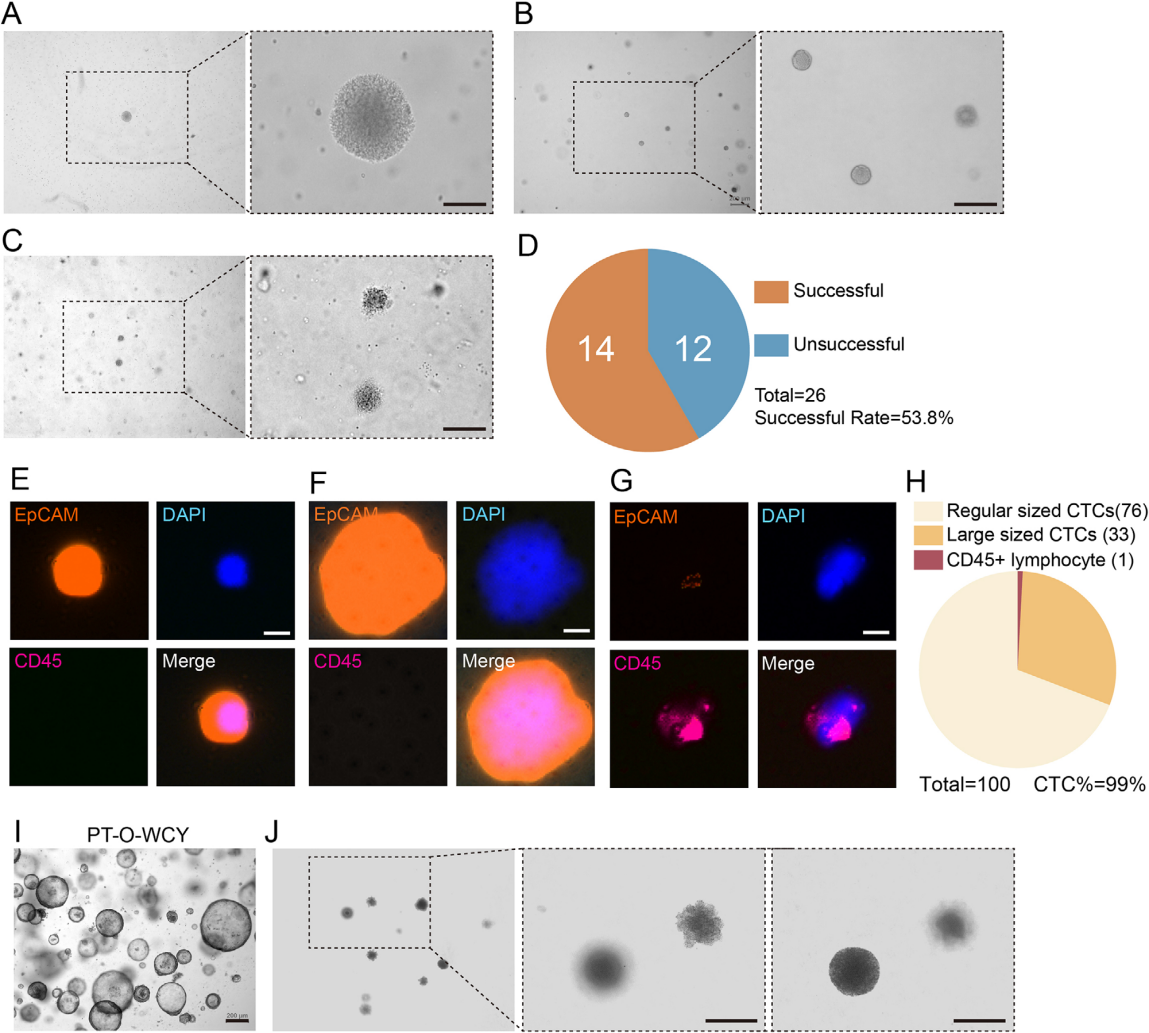
The culture and identification of circulating tumor cell organoids. (**A**) Passage 1 CTC organoid observed 14 days after seeding. (**B**) Passage 2 CTC organoids observed 14 days after re‐seeding. (**C**) Passage 3 CTC organoids observed 30 days after re‐seeding. Scale bars = 100μm in (**A)**–(**C)**. (**D**) CTC organoids were cultivated successfully from 14 out of 26 blood samples derived from 26 different patients, making the successful rate 53.8%. (**E**) A regular‐sized CTC was stained DAPI positive, EpCAM positive, and CD45 negative. (**F**) A large‐sized CTC was stained DAPI positive, EpCAM positive, and CD45 negative, which has a large nucleus and large cytoplasm. (**G**) A lymphocyte was stained DAPI positive, EpCAM negative, and CD45 positive. (**H**) Of the 100 cells detected, there were 76 regular‐sized CTCs, 33 large‐sized CTCs, and one lymphocyte. Scale bars = 10 μm in (**E)**–(**G)**. (**I**) Organoids derived from a patient's primary tumor (PT‐O‐WCY). Scale bar = 200 μm. (**J**) Second passage CTC organoids derived from the same patient cultured for over 5 months exhibit different morphologies. Scale bars = 20 μm.

To test whether the observed organoids are composed of CTCs, CTC organoids were harvested, dispersed, washed by PBS for twice, and resuspended by intestinal organoid culture medium. The cell suspension was delivered to Shanghai Epione Medlab (a Dunwill Company) for the detection of CTCs and lymphocytes using ChimeraX^®^ CTC detection system developed by Genovo (Dunwill Company), which uses wide field imaging system to capture fluorescence. EpCAM was used as a maker for CTCs, and CD45 was used as a marker for lymphocytes. Almost all the cells showed positive staining of EpCAM (orange), positive staining of DAPI (blue), and negative staining of CD45 (magenta), while a lymphocyte showed positive stainings of CD45 and DAPI, and negative staining of EpCAM (Figure 2E–G). Notably, the detection revealed that there are two types of CTCs in the organoids. One type of CTCs showed a regular cell size (Figure 2E), while the other showed a large cell size, with both a large nucleus and large cytoplasm (Figure 2F). In 100 CTCs we detected, 76 cells were regular‐sized, 33 cells were large‐sized, and one lymphocyte was also detected (Figure 2G). Thus, the detection results showed that the organoids we observed were composed of at least 99% CTCs, indicating that the organoids we cultured were indeed CTC organoids.

In contrast to the organoids derived from patients’ primary tumors (Figure 2I, PT‐O‐WCY), CTC organoids showed a unique morphology (Figure 2A–C and J). Interestingly, after cultivating the organoids in Figure 2C for 5 months, we observed CTC organoids derived from the same patient showed distinct morphologies (Figure 2J). One of the organoids still showed a subsphaeroidal shape (Figure 2J, right), while the other showed a shape resembling a young dandelion with petals, hinting that the organoids might be differentiated (Figure 2J, middle). As we observed, CTC organoids differ from organoids derived from primary tumors and exhibit heterogeneity among organoids derived from the same patient, and among the cells composing CTC organoids.

In conclusion, we successfully cultured and identified CRC CTC organoids derived from the blood of mCRC patients, and this technique might serve as a primary basis for a model facilitating personalized therapy for mCRC patients suffering from unresectable metastases.

## Funding information

Science and Technology Commission of Shanghai Municipality; Grant Number: 18401933402 (to Peng); Shanghai Sailing Program; Grant Number: 19YF1409500 (to Li); Shanghai Anticancer Association EYAS PROJECT; Grant Number: SACA‐CY1A05 (to Li); National Natural Science Foundation of China; Grant Number: 81672374 (to Cai); National Natural Science Foundation of China; Grant Number: 81972244 (to Hu); National Natural Science Foundation of China; Grant Number: U1932145 (to Peng).

## CONFLICT OF INTEREST

The authors declare that there is no conflict of interest.

## ETHICS APPROVAL

The research was approved by the Ethical Committee and Institutional Review Board of Fudan University Shanghai Cancer Center.

## AUTHOR CONTRIBUTIONS

Long Zhang, Yaqi Li, and Junjie Peng designed the study; Long Zhang performed most of the experiments; Long Zhang, Yaqi Li, and Junjie Peng analyzed the data and co‐wrote the manuscript. Shaobo Mo, Xiang Hu, and Xiaoji Ma participated in some experiments; Shaobo Mo and Xiang Hu co‐wrote the manuscript and organized the figures. Weixiang Jin and David Haixiang Peng participated in CTC identification experiments, Yu Zhou and Xiaofei Liang provided essential support to this study. Xinxin Rao, Xiaoya Xu, Zhen Zhang, and Guoqiang Hua provided support and suggestions for this study. Sanjun Cai and Junjie Peng conceptualized, designed, and directed the project. All authors read and approved the manuscript.

## Supporting information

SUPPORTING INFORMATION
