# Supplementary material for: Establishment and identification of organoids from human circulating colorectal cancer cells
Source: Clin Transl Med. 2020 Dec 12;10(8):e247. doi: 10.1002/ctm2.247 (PMC7733316; doi:10.1002/ctm2.247)
Supplement: Supplementary file 1 — SUPPORTING INFORMATION [file CTM2-10-e247-s001.docx]

| Table S1. Patient clinicopathological information | | | | | | | |
| --- | --- | --- | --- | --- | --- | --- | --- |
| Patient IDs | Age at diagnosis | Gender | Pre-CEA | pT | pN | M | Metastasis site |
| CRC-001 | 62 | Male | Positive | 4 | 2 | 1 | Liver |
| CRC-002 | 64 | Male | Positive | 3 | 1 | 1 | Liver |
| CRC-003 | 51 | Male | Negative | 4 | 2 | 1 | Lung |
| CRC-004 | 62 | Male | Positive | 4 | 2 | 1 | Liver |
| CRC-005 | 71 | Male | Positive | 3 | 2 | 1 | Lung |
| CRC-006 | 77 | Male | Positive | 2 | 0 | 1 | Liver |
| CRC-007 | 69 | Male | Positive | 3 | 1 | 1 | Liver, Lung |
| CRC-008 | 66 | Male | Negative | 3 | 1 | 1 | Liver |
| CRC-009 | 59 | Female | Positive | 3 | 0 | 1 | Liver, Lung |
| CRC-010 | 69 | Female | Positive | 3 | 0 | 1 | Liver |
| CRC-011 | 66 | Male | Positive | 3 | 0 | 1 | Lung |
| CRC-012 | 71 | Female | Positive | 4 | 0 | 1 | Liver |
| CRC-013 | 35 | Female | Positive | 3 | 2 | 1 | Lung |
| CRC-014 | 43 | Female | Positive | 4 | 2 | 1 | Lung |
| CRC-015 | 54 | Female | Positive | 2 | 0 | 1 | Liver |
| CRC-016 | 65 | Female | Positive | 3 | 2 | 1 | Liver |
| CRC-017 | 44 | Female | Negative | 3 | 0 | 1 | Bone |
| CRC-018 | 45 | Female | Negative | 3 | 2 | 1 | Peritoneum |
| CRC-019 | 65 | Female | Negative | Unknown | Unknown | 1 | Liver, Peritoneum |
| CRC-020 | 65 | Female | Negative | 4 | 1 | 1 | Liver |
| CRC-021 | 67 | Male | Negative | 4 | 1 | 1 | Liver |
| CRC-022 | 67 | Female | Negative | 2 | 1 | 1 | Peritoneum, Bone |
| CRC-023 | 80 | Male | Positive | 3 | 1 | 1 | Liver |
| CRC-024 | 66 | Male | Positive | 1 | 0 | 1 | Liver |
| CRC-025 | 62 | Female | Positive | 3 | 1 | 1 | Liver |
| CRC-026 | 61 | Male | Negative | 2 | 0 | 1 | Liver |
| Pre-CEA, Pretreatment carcinoembryonic antigen | | | | | | | |

| Table 1 Patient clinicopathological information, continued | | | | | | |
| --- | --- | --- | --- | --- | --- | --- |
| Tumor site | Histology | KRAS status | NRAS status | BRAF status | MMR status | Success |
| Left | AD | Unknown | Unknown | Unknown | Unknown | Yes |
| Left | AD | WT | MT | WT | pMMR | No |
| Right | MAD | WT | WT | WT | dMMR | No |
| Left | AD | WT | WT | WT | Unknown | Yes |
| Left | AD | WT | WT | WT | pMMR | Yes |
| Left | AD | MT | WT | WT | pMMR | No |
| Left | AD | Unknown | Unknown | Unknown | pMMR | No |
| Left | AD | WT | WT | WT | pMMR | No |
| Left | AD | WT | WT | WT | pMMR | Yes |
| Right | AD | MT | WT | WT | pMMR | No |
| Right | AD | Unknown | Unknown | Unknown | pMMR | Yes |
| Right | AD | MT | WT | WT | pMMR | No |
| Left | AD | MT | WT | WT | pMMR | No |
| Left | AD | WT | WT | WT | pMMR | No |
| Right | AD | Unknown | Unknown | Unknown | pMMR | No |
| Left | MAD | MT | WT | WT | pMMR | No |
| Left | AD | WT | WT | MT | pMMR | Yes |
| Right | MAD | WT | WT | MT | Unknown | No |
| Right | AD | WT | WT | MT | Unknown | Yes |
| Right | AD | WT | WT | MT | pMMR | Yes |
| Right | AD | WT | MT | WT | pMMR | No |
| Left | AD | WT | WT | MT | pMMR | No |
| Left | AD | MT | WT | WT | pMMR | Yes |
| Left | AD | MT | WT | WT | pMMR | No |
| Left | AD | WT | WT | WT | pMMR | Yes |
| Left | AD | Unknown | Unknown | Unknown | Unknown | Yes |
| AD, Adenocarcinoma; MAD, Mucinous adenocarcinoma; WT, Wild type; MT, Mutant type; MMR, Mismatch repair; pMMR, proficient mismatch repair; dMMR, deficient mismatch repair | | | | | | |
